# Are We Ready for It? A Review of Forensic Applications and Readiness for Comprehensive Two‐Dimensional Gas Chromatography in Routine Forensic Analysis

**DOI:** 10.1002/jssc.70138

**Published:** 2025-04-21

**Authors:** Emma L. Macturk, Kevin Hayes, Gwen O'Sullivan, Katelynn A. Perrault Uptmor

**Affiliations:** ^1^ Chemistry Department, William & Mary Nontargeted Separations Laboratory Williamsburg Virginia USA; ^2^ Environmental Forensics and Arson Laboratory Department of Earth and Environmental Science Mount Royal University Calgary Canada

## Abstract

Comprehensive two‐dimensional gas chromatography (GC×GC) has been explored in forensic research to provide advanced chromatographic separation for forensic evidence, including illicit drugs, fingerprint residue, chemical, biological, nuclear, and radioactive (CBNR) substances, toxicological evidence, odor decomposition, and petroleum analysis for arson investigations and oil spill tracing. In GC×GC, the separation and analysis of analytes is similar to one‐dimensional GC, but the primary column is connected to a secondary column via a modulator to provide two independent separation mechanisms, thus increasing the peak capacity of the analysis. The goal of implementing GC×GC in forensic studies is often to increase the separation and detectability of analytes and has most often been applied in nontargeted forensic applications where a wide range of analytes must be analyzed simultaneously. To date, there has been no summary of the current state of forensic research that evaluates both analytical and legal readiness for routine use. For these analytical methods to be adopted into forensic laboratories and be used in evidence analysis, they must meet rigorous analytical standards. In addition, new analytical methods for evidence analysis must adhere to standards laid out by the legal system, including the Frye Standard, Daubert Standard, and Federal Rule of Evidence 702 in the United States and the Mohan Criteria in Canada. Current research on GC×GC use for forensic applications was summarized and reviewed for analytical advances and technology readiness to provide a comprehensive view of GC×GC use for future routine implementation. A technology readiness scale, with levels from 1 to 4, was used to characterize the advancement of research in each individual application area. Seven forensic chemistry applications are discussed related to courtroom criteria and categorized into technology readiness levels based on current literature as of 2024. Future directions for all applications should place a focus on increased intra‐ and inter‐laboratory validation, error rate analysis, and standardization.

## 1 Introduction

Comprehensive two‐dimensional gas chromatography (GC×GC) is an analytical technique used to separate volatile and semi‐volatile compounds in complex mixtures. GC×GC expands upon the traditional separation technique of one‐dimensional gas chromatography (1D GC) by adjoining two columns of different stationary phases in series with a modulator [1]. Although 1D GC methods have limitations on resolution and detectability for trace compounds, GC×GC offers an increase in signal‐to‐noise ratio and overall larger peak capacity that enables more comprehensive separation of complex samples [2, 3]. The modulator, commonly referred to as the heart of GC×GC, preserves the separation from the first column by sending a short retention time window to be separated on the secondary column. Modulation allows for the analytes’ different affinities for each column to dictate their separation and increase overall peak capacity.

**FIGURE 1:**
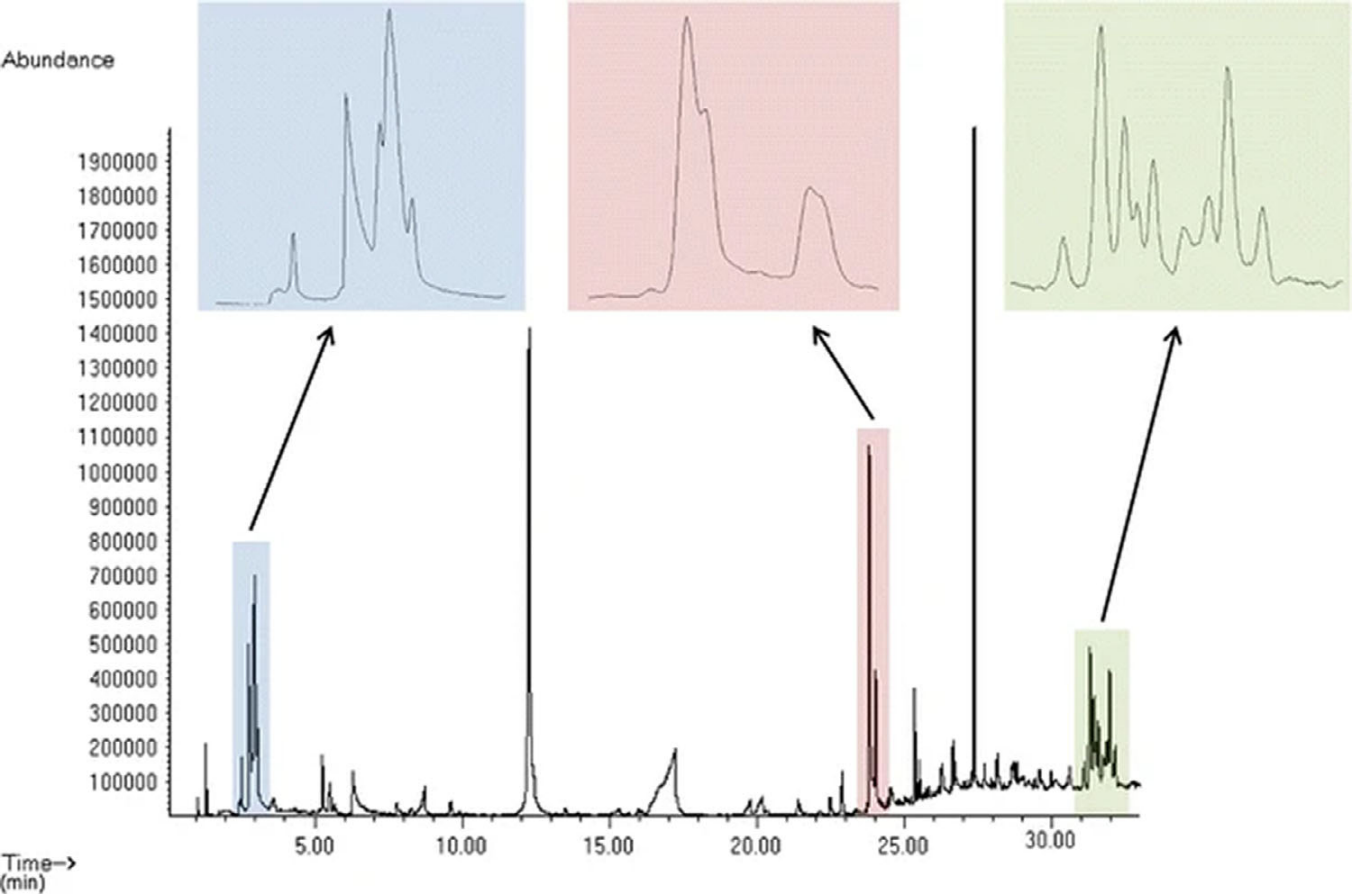
One‐dimensional GC total ion chromatogram showing co‐elutions of volatiles from decomposition of remains [8].

**FIGURE 2:**
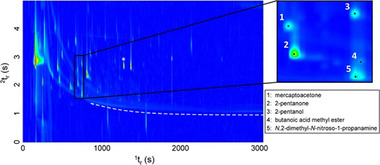
GC×GC‐TOFMS total ion chromatogram demonstrating the resolving power of multidimensional separations [101].

Multidimensional gas chromatography has developed and evolved since its conception in the 1980s. The need for better separation and sensitivity in complex samples has driven the evolution of GC×GC since it was first introduced [4, 5, 6, 7]. Theory development pioneered in the 1980s was driven by the need for improved peak capacity [4, 5]. Figures 1 and 2 demonstrate the ability of GC × GC to resolve analytes that co‐elute in 1D GC [8]. The first demonstrated success of GC×GC was published in 1991, resolving a 14‐component, low‐molecular‐weight mixture [6]. The first formal definitions in the field were published in 2003 [9] and then updated in 2012 [7].

In GC×GC, a sample is injected onto a primary column (^1^D column) of a desired stationary phase. The analytes within the sample elute from the ^1^D column according to their affinity for its stationary phase [1, 2, 3]. The modulator collects samples from the eluate for a set time period (e.g., typically 1–5 s) and then passes these collected plugs onto the secondary column (^2^D column) at a repeated interval, called the modulation period [1, 2, 3]. The ^2^D column separates the short injection from the modulator further, according to a different retention mechanism than the ^1^D column. Detectors for GC×GC have evolved from early detection methods using flame ionization detection (FID) and mass spectrometry (MS) to more advanced methods including high‐resolution (HR) MS and time‐of‐flight (TOF) MS, as well as dual detection methods such as TOFMS/FID [10].

**FIGURE 3:**
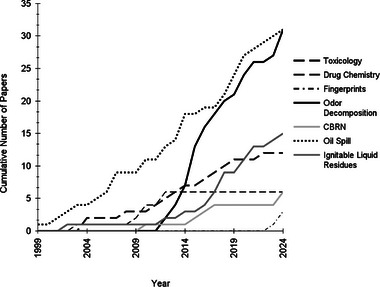
Cumulative number of works per year up to 2024 in seven forensic chemistry applications using comprehensive two‐dimensional gas chromatography.

Current applications of GC×GC use its high peak capacity in fields, such as fuels and industrial chemicals, environmental analysis, foods and fragrances, and biological and forensic studies [1]. The technique of GC×GC was pioneered in 1991 and has developed greatly in the field of separation chemistry in terms of hardware, method development, and data processing over the past 30+ years [6]. Early applications from 1999 to around 2012 focused on proof‐of‐concept studies for forensic applications of GC×GC and then rapidly increased in number (Figure 3). In recent years, the areas of oil spill forensics and decomposition odor as forensic evidence have reached 30+ works for each application (Figure 3). This trend demonstrates a growing interest and wider acceptance of GC×GC in the forensic sphere.

Current literature reports forensic usage of GC×GC–MS to characterize drugs [11, 12, 13, 14, 15]; forensic toxicology [16, 17, 18]; fingermark chemistry [19, 20, 21]; odor decomposition [22, 23, 24, 25, 26, 27, 28]; chemical, biological, nuclear, and radioactive (CBNR) forensics [29, 30, 31, 32, 33, 34]; and environmental forensics concerning ignitable liquid residue (ILR) [2, 35, 36, 37, 38, 39, 40] and oil spill tracing [41, 42, 43, 44, 45, 46, 47, 48, 49, 50, 51]. Volatilome profiling of living human odor and illegal wildlife items has been briefly investigated [52, 53]; the technique, however, is not currently used extensively in those two applications and will not be touched on in this review. Common trace evidence materials, including drug chemistry, toxicology, and CBNR materials, are gaining more attention for analysis using GC×GC. Routine evidence analysis in forensic science laboratories does not currently use GC×GC–MS as an analytical technique due to strict criteria set by legal systems that limit the entrance of scientific expert testimony into a legal proceeding. This can be a challenge for analytical chemists working on new method development in forensic research applications, because the standards required of research for eventual admission into the legal system are not set by scientists but rather other stakeholders in the legal system. In the United States, the Frye and Daubert Standards followed by individual states laid the groundwork for the Federal Rule of Evidence 702 to guide admissibility of expert testimony [54, 55, 56]. In Canada, the Mohan criteria set the groundwork for relevance and necessity for evidence admission to court [57]. These three court cases, which will be discussed relevant to the GC×GC transition into routine analysis, play a key role in setting precedent for which analytical chemistry techniques are acceptable in forensic laboratories.

This review will evaluate current and past literature on forensic chemistry applications using GC×GC. Each application of forensic chemistry using GC×GC will be examined and assessed for its analytical readiness, technological readiness, and legal readiness for use in routine casework.

## 2 Courtroom Implications

As analytical chemists, it is important to be mindful of the end use of new analytical developments and ensure they are fit‐for‐purpose. Legal benchmarks must be met in this applied area of analytical chemistry. GC–MS methods have been considered “gold standard” in forensic laboratories for use in expert testimony; as such, a transition to GC×GC–MS would provide reliable forensic evidence in a courtroom.

Court systems have preceding rules for the admission of expert testimony to be used as evidence in a trial. In 1923, the standard of evidence admissibility was first set in the case of *Frye v. United States*, where the federal court ruled that expert testimony on a scientific technique should only be admitted as evidence if the technique was “generally accepted in the relevant scientific community” [54]. Since the “general acceptance” requirement was vague, the case of *Daubert v. Merrell Dow Pharmaceuticals, Inc*. (1993) expanded on this through providing an “appropriate validation” process [55]. This is based on the judge as gatekeeper assessing four factors, including whether (1) the technique can or has been tested, (2) the technique has been peer‐reviewed and published, (3) there is a known rate of error or methods of controlling error, and (4) the theory or technique is generally accepted (Table 1) [54, 55]. The Daubert Standard has been used across federal courts since the 1993 case and has been adopted in the Federal Rule of Evidence 702 (Fed. R. Evid. 702) statute as an amendment in 2000 [56]. State courts may choose to follow either standard. The principle of general acceptance in a scientific community consistently relies on the utility and reliability of a technique or method, so analytical techniques must be thoroughly supported prior to adoption in legal proceedings.

**TABLE 1:** Rules for the admissibility of evidence in four court cases in the United States and Canada.

Frye	Daubert	Mohan	Federal rule of 702
General acceptance	General acceptance in relevant scientific community		
	Whether the theory/technique has been peer reviewed and published		Testimony has sufficient facts or data to support
	Whether the theory/method has been or can be tested		
	Whether there is a known error rate within acceptable limits		Testimony produced from reliable principles and methods
		Necessity of the expert in helping the jury understand the evidence	Expert's knowledge aids in understanding the evidence
		Absence of exclusionary rules	
		The expert testifying is properly qualified	The expert has applied appropriate principles and methods to the case
		Relevance to the case	

In Canada, *R. v. Mohan*, [1994] 2 S.C.R. 9 (Can.) ruled that expert evidence is admitted on the basis of the following four factors: (1) its relevance to the case; (2) the necessity of the evidence in assisting the trier of fact; (3) the absence of any exclusionary rule applied to the evidence; and (4) a properly qualified expert is testifying [57]. The *Mohan* criteria states that expert evidence is “subjected to special scrutiny to determine whether it meets a basic threshold of reliability” [57]. The theme of scientific expert testimony reliability in the United States and Canada is clearly stated numerous times in the court reports and dissents on the subject. Table 1 represents the various benchmarks that new technology must meet. These criteria cover aspects of method development in an analytical chemistry laboratory; sufficient data, reliable methods, and known error rates imply method development and optimization, including figures of merit, intra‐ and inter‐laboratory validation, and standardization of methods. These essential processes progress new analytical techniques from discovery to routine use.

In addition, the expert must be evaluated through *voir dire*, where the individual will be questioned by both the prosecution and the defense to understand their qualifications, education, and training related to the expert testimony in question [56]. Juries must be able to comprehend and make an informed judgment pertaining to the case based on the evidence presented by expert witnesses. A recently published study found that lay audiences were equally likely to be able to identify similarities and differences between 1D and 2D chromatograms and that 2D chromatograms were rated no more difficult to distinguish than 1D chromatograms [58]. Through *voir dire*, the expert in question is assessed on the basis of sufficient data, reliable methods, and appropriately applied methods to the case in question [56]. Before the testimony by an expert forensic witness can be admitted in legal proceedings, the technique they use for evidence analysis must be developed to be accessible in forensic laboratories. This provides emphasis on the need for a competent workforce and training pipeline when new techniques are introduced into forensic practice.

To translate techniques from novel to routine use in forensic laboratories, a system measuring technology readiness level (TRL) is common in forensic chemistry research. For example, the *Forensic Chemistry* journal uses TRLs to assign techniques, methods, or ideas based on the ease of implementation into the operation of a forensics laboratory [59]. Ideas, technologies, or methods are assigned to four TRLs (Table 2). Other organizations and disciplines also use similar scales. NASA, for example, uses a TRL scale with nine levels that follow a similar pattern where TRL 1 consists of basic principles and TRL 9 technologies have successfully completed mission testing [60]. The highest TRL of 4 in forensic chemistry research can be considered coinciding with the relevant and reliable criteria for admission of expert testimony using GC×GC to analyze forensic evidence.

**TABLE 2:** Criteria for technology readiness levels in forensic chemistry, adapted from the journal Forensic Chemistry [59].

Level 1	Basic research phenomenon observed
	Basic theory proposed
Level 2	Development of a theory that has a demonstrated application to a specified area of forensic chemistry
	Development of research phenomenon that has a demonstrated application to a specified area of forensic chemistry
	Supporting data
Level 3	Application of an established technique or instrument to a specified area of forensic chemistry
	Some measurement of uncertainty or error
	Developed aspects of intra‐laboratory validation through measurement of figures of merit (e.g., …)
	Methods should be practicable on commercially available instruments
	Communication and results of the first inter‐laboratory trials
Level 4	Refinement, enhancement, and inter‐laboratory validation of a standardized method ready for implementation in forensic laboratories
	New knowledge in this area can be immediately adopted or used in casework

The adaptation of new analytical techniques in forensic laboratories will serve courtrooms as long as there has been rigorous analytical method development and expansion upon GC×GC in relevant application fields.

## 3 Fingerprint Chemistry

Ridge characteristics from a deposited fingerprint are routinely used by forensic identification specialists to individualize a fingerprint to a suspect via the use of a fingerprint database. Partial fingerprints, or fingermarks, consisting of sweat and oil that are smudged or lacking a complete ridge pattern are not fit to undergo the identification process, although their chemical residue could still be useful to forensic chemists [61]. Though GC–MS has been used to characterize components of this residue, fingermark analysis using GC×GC–MS is in its infancy. Established GC–MS studies can determine donor class characteristics such as sex or relative age of a donor using biomarkers of octadecanol (C_18_) and eicosanol (C_20_) for sex determination and squalene and cholesterol for relative aging [62, 63]. Three manuscripts were identified to use GC×GC–MS for chemical analysis of fingerprint residue.

One proof‐of‐concept study for GC×GC–MS analysis of fingermarks was performed by Ripszam et al., in which the group used GC×GC‐HRMS to identify 326 compounds in human sweat, one of the substituents of fingermark residue [20]. Of these compounds, 239 originated in the human body, and 41 were identified as anthropogenic, or originating as a contaminant outside the body [20]. Two studies directly analyzed fingerprint residue using GC×GC‐TOFMS; one targeted method was performed for identifying seven analytes, and one study with focused on the extraction of residue from fired cartridges for exploiting chemical differences within suspects [19, 21]. Given the large peak capacity of GC×GC and the ability to perform nontargeted analysis of complex samples with large numbers of analytes, GC×GC performance in the former study was not exploited to its potential extent [19]. The second study investigated fingerprint residue from fired gun cartridges and demonstrated proof‐of‐concept work to differentiate residue from five volunteers in a forensic case setting [21]. These proof‐of‐concept studies could be extrapolated to fingermark profiling with applications for analyzing forensic evidence. Further analysis of fingermark residue in a nontargeted manner could improve the ability for trace compound detection within fingermark residue.

These preliminary studies are the first of their kind in this application to use GC×GC–MS to analyze their respective sample type within a forensic context. Additionally, these studies were published in 2023 and 2024; this recent interest in characterizing biological sebaceous excretions using GC×GC–MS expands research applications for GC×GC–MS in the forensic chemistry field. Several factors of the Daubert and Mohan criteria are not met by this application of forensic analysis. This application of two published studies does not lead to qualified expert witnesses available for testimony. Although one study addressed error rates of intra‐ and inter‐sample variability, there are no known or acceptable limits for this application. Method development and additional supporting data are necessary to move this application beyond a TRL of 1.

## 4 Chemical, Biological, Nuclear, and Radioactive

The application of GC×GC for the analysis of chemical weapons and explosives is in early stages of development. Eight manuscripts were identified that examined the application; of those, five used chemical weapon or explosive samples [30, 33, 34, 64, 65], with the remaining three employing proxy or precursor compounds [29, 31, 32]. Sample sizes were small for all studies with the exception of Strozier et al. and Wang et al.; the aforementioned consisted of less than 10 samples (not including replicates of the same sample) in each study [31, 33].

The authors of the respective studies explicitly describe the maturity of the use of GC×GC–MS for chemical weapons and explosives applications. Reichenbach et al. is the earliest identified manuscript that discusses the separatory enhancement of GC×GC–MS relating to non‐chemical weapon applications [64]. Stefanuto et al. and Strozier et al. describe the work completed as proof‐of‐concept [30, 31]. Tsai et al., Gravett et al., and Lu et al. describe their work as preliminary investigations [32, 34, 65]. Wang et al. emphasizes issues related to the practicality of applications due to limited data and a need for replication [33]. Hoggard et al. suggests that the research completed in the study is novel and aims to “demonstrate the feasibility” of their process [29]. The authors’ descriptions of their respective work in this application have collective themes of novelty and exploratory focus to provide foundations for expansion of these topics in the future.

Two studies noted direct comparisons with 1D GC. In both GC×GC separation coupled with TOFMS, noted advantages over 1D GC. In Gravett et al., GC×GC‐TOFMS was used to supplement 1D GC–MS coupled with flame photometric detection (FPD) and LC–HRMS [65]. The authors list 17 compounds detected and identified in crude VX utilizing gas chromatographic methods; of those, 10 were exclusively identified via GC×GC–TOFMS and one was identified solely by 1D GC [65]. Stefanuto et al. utilized fast GC–TOFMS and GC×GC–TOFMS to identify the volatile compound signature of explosives; fast 1D GC proved to have insufficient separation capacity during method optimization, and thus the GC×GC separation technique was selected for the completed analysis [30]. All other manuscripts within this section exclusively used GC×GC separation.

Despite the advantages demonstrated, further research is required to establish the readiness of the separation method for this application. Thus far, the available research suggests that the chromatographic techniques employed in each study are replicable and have met the rigor of peer review. However, there is no evidence of replicated results, no established error rates, nor any inter‐laboratory studies that would allow for a designation at a higher TRL level. Likewise, the only evidence of an established method being employed is the use of an “FBI Protocol” for sample extraction in Tsai et al. [32] As such, for this application, GC×GCanalysis of CBNR evidence is unlikely to meet the Daubert Standard and could be assessed a TRL of 1.

## 5 Drug Chemistry

This review identified nine manuscripts in which GC×GC–MS was utilized for the assessment of drugs [11, 13, 14, 15, 66, 67, 68, 69, 70]. In each of these studies, an analysis of illicit drugs or potential drugs of abuse is conducted. These works emphasized the utility of GC×GC–MS to chromatographically separate mixtures of illicit drugs or to complete impurity assessments of the drugs themselves.

Early drug assessments using GC×GC–MS include two Song et al. studies [66, 67]. The authors examined standardized mixtures of drugs, utilizing GC×GC–TOFMS and GC×GC–quadrupole MS (qMS), and then compared both methods to conventional GC–MS and each other. Although the qMS detector was largely too slow for the application of the analytical technique, the GC×GC–TOFMS assessment, which included four genuine forensic samples of whole blood, demonstrated promising results [66]. Sample preparation and methodology issues, such as the non‐derivatization of the drugs included in the samples, are described as hampering the detection of several drugs [66, 67]. GC×GC–qMS was also utilized by Omar et al. [15]. In this small study, 17 samples of buds and leaves from five cannabis strains were analyzed. The authors were able to improve the resolution issues associated with the detector with statistical techniques [15]. These early application studies set a foundation for future studies, which are further along the TRL scale.

In a proof‐of‐concept study, Gröger et al. conducted a chemical fingerprinting assessment of cannabis, cannabis resin, and heroin [11]. This group adapted established and harmonized GC methods to GC×GC–TOFMS for the assessment of heroin, but no such method existed for the cannabis tested. This study was followed by three subsequent impurity assessments within the Zimmermann laboratory group of methylenedioxymethamphetamine (MDMA), damiana (a common substrate for synthetic cannabis), and heroin [68, 69, 70]. Schäffer et al. utilized and adapted a harmonized gas‐chromatographic method (CHAMP) [69]; Gröger and Schwemer utilized similar extraction techniques but different column sets and instrument conditions [11, 68]. These studies demonstrate successful application of GC×GC–TOFMS to illicit drug detection for these particular drugs.

Knorr et al. and Muthal and Snow both describe applications for GC×GC–TOFMS for non‐illicit drugs that could potentially be abused (cigarettes and nonsteroidal anti‐inflammatory drugs, respectively) [13, 14]. These studies both detail the successful analysis of compounds that are generally unsuited for conventional GC–MS and GC×GC–TOFMS techniques due to low volatility; the analysis described would routinely be carried out via liquid chromatography (LC) techniques. Knorr et al. provide the first mention of utilizing a nontargeted analysis for this application using derivatization [13]. However, there is a non‐forensic focus for both of these studies, likely due to the lawful and permissible uses of the products assessed. Research on the abuse of these drugs, however, could be of forensic interest after applied use of GC×GC–TOFMS for analysis.

Some of the research included in drug assessment and analysis by GC×GC–MS has adapted conventional GC–MS methods, which have been established in harmonized inter‐laboratory settings [11, 69]. Several of the works describe a comparison between the conventional and established GC–MS and GC–HRMS techniques and emphasize the utility of multidimensional chromatography in these settings [66, 67, 68, 69, 70]. These findings all provide support for the reliability of GC×GC–MS as a technique and ability to separate and identify drugs and drug impurities. However, no manuscript described inter‐laboratory studies for the specific GC×GC techniques and this application. Likewise, the description of method accuracy, repeat analysis, and sufficient sample size is limited. As such, the application lacks the validation that would provide for more complete general acceptance. This positions the application of GC×GC for the analysis of drugs and drug impurities at a TRL of 2.

## 6 Toxicology

To the contrary of the field of forensic drug chemistry that investigates drugs as physical evidence outside the body, the field of forensic toxicology focuses on the analysis of drugs from human specimens, either as the native drug molecule or as drug metabolites. Toxicology research often focuses both on samples from living individuals (often from blood, urine, or saliva samples) and on samples from deceased individuals through postmortem toxicology (often involving a wider range of sample specimens). Six works focusing on applications of forensic toxicology using GC×GC were identified in the literature, ranging from 2003 to 2018, which represented a broad range of proof‐of‐concept applications in various drug categories [16, 17, 18, 71, 72, 73].

LC, mostly in combination with various MS approaches, has largely been the main tool used for confirmatory analysis in forensic toxicology. However, there has been increasing interest in the nontargeted screening nature of GC×GC–TOFMS for its expanded ability to perform high‐throughput screening of a wide array of analytes in a single method [16]. Another major benefit of this technique is the ability to physically resolve endogenous analytes within the matrix from exogenous compounds due to drug ingestion [18]. Early studies in forensic toxicological analysis using GC×GC–MS focused on the technique in application to anti‐doping efforts as an analytical alternative to LC techniques. Several basic drugs, as well as opiates, opioids, cocaine, and benzodiazepines, were shown to be capable of analysis using GC×GC–MS methods so long as derivatization was applied to the sample in advance of injection [17, 18]. This derivatization step is important to make the analytes more amenable to gas chromatographic separation. Twelve psychoactive drugs, including MDMA, ketamine, and cocaine, were effectively analyzed without derivatization using GC×GC‐FID recently and validated using Standard Practices for Method Validation in Forensic Toxicology guidelines set by the American Academy of Forensic Sciences (AAFS) [74, 75]. In the application of anti‐doping, GC×GC‐TOFMS has also been shown to reach the required sensitivity for the guidelines put in place by the World Anti‐Doping Agency (WADA) for at least 40 analytes of interest and for improved full‐scan monitoring of certain drugs of interest, such as hydroxystanozolol, that are particularly challenging to detect without selected ion monitoring [18, 71]. The ability to detect toxicological evidence in a nontargeted manner is an advantage of using GC×GC for this purpose over LC.

Aside from doping agents, GC×GC has also been applied to other drugs of interest. Proof‐of‐concept studies have shown potential for eight different drugs in hair samples [16]. Validation studies have demonstrated the possibility of simultaneous detection of cannabinoids extracted from plasma and blood [72, 73]. The literature basis indicates a TRL of 1 due to the lack of repeated literature, inter‐laboratory studies, lack of uncertainty and error reporting, and lack of supporting data through additional publications. Due to the large number of analytes being monitored in forensic toxicology, more supporting data are needed to move this field toward TRL 2. The validation studies on cannabinoids are a promising direction for drug monitoring as it begins to approach some concepts seen in TRL 3. Anti‐doping research comparison to world standards set by WADA and quality validation by methods guidelines set by the AAFS also provides some promise toward the advancement of this application in terms of its TRL. Nevertheless, an increase in more recent work across the variety of drug analytes observed in forensic toxicology would be a valuable addition to the field. In terms of the Daubert criteria, although the technique has been peer‐reviewed, published, and tested in some scenarios, it is not yet generally accepted, and error rates have not been characterized. Additional work should aim to assist in furthering the TRL scale through supporting data and error rate characterization to lend additional validity to the method for forensic toxicology analyses.

## 7 Ignitable Liquid Residue

The identification and characterization of ILR in fire debris are critical steps in arson investigations to determine whether an accelerant was used and, if so, what kind. Fire debris analysis is particularly challenging due to the variability of substrates, presence of interfering compounds from pyrolysis, complexity of ignitable liquids, and often trace‐level presence of these liquids due to consumption by fire, weathering, and environmental factors [39].

To date, gas chromatography coupled with either FID (GC‐FID) or MS (GC–MS) has been the primary method for analyzing fire debris samples in arson cases following ASTM International (ASTM) protocols [76]. However, challenges related to sensitivity and separation have prompted the exploration of more advanced methods, including the use of GC×GC. Phillips et al. presented the first application of GC×GC for ILR analysis [77], but further development was limited until the early 2000s when Frysinger and Gaines recognized the potential of GC×GC for fire debris analysis, securing a grant from the National Institute of Justice (USA) to validate the technique [78]. These two early studies observed basic research phenomena using GC×GC in this field.

**FIGURE 4:**
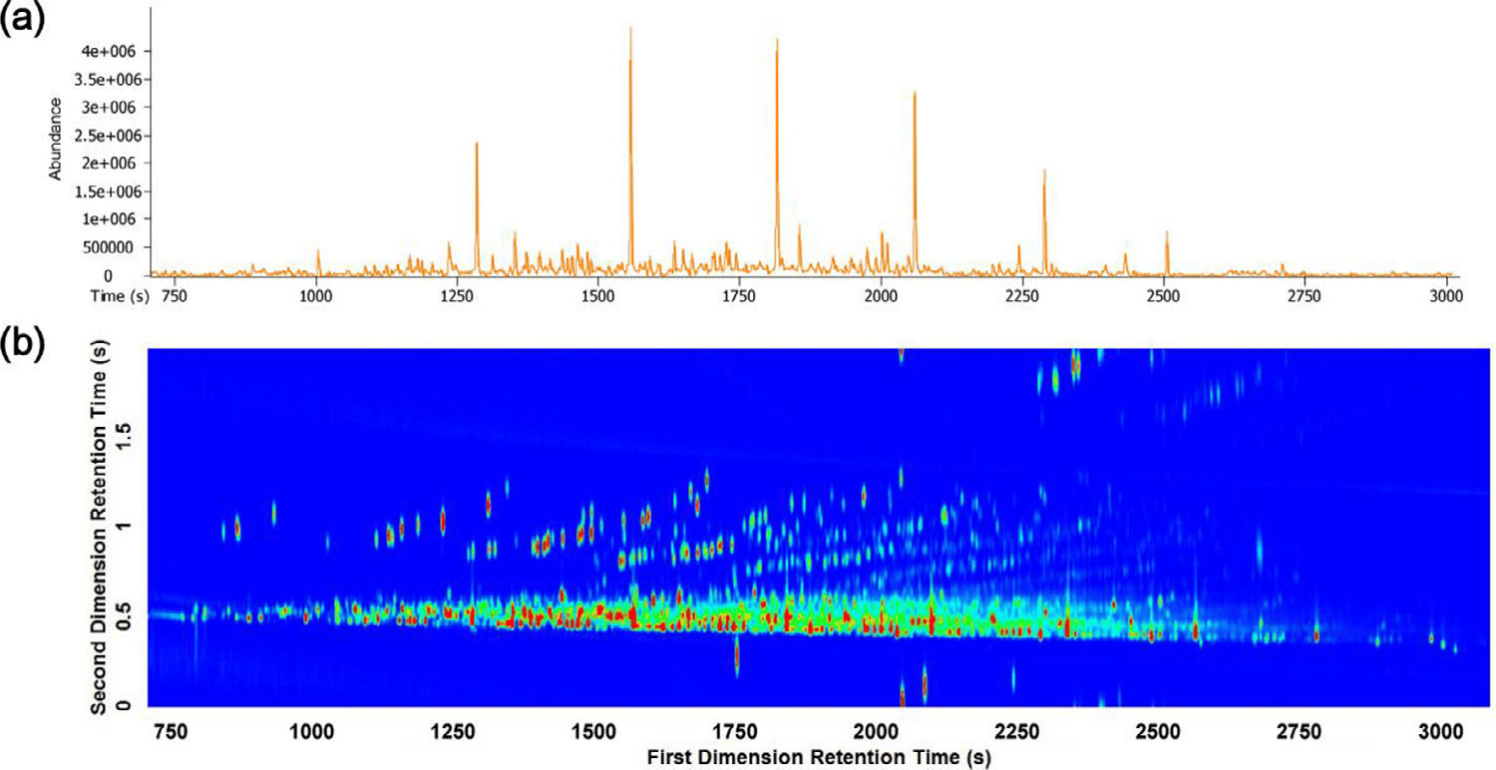
One‐dimensional (a) and two‐dimensional (b) chromatograms of kerosene [82].

Subsequent studies investigated the use of GC×GC coupled with FID, qMS, and TOFMS for ILR analysis in fire debris, demonstrating improved sensitivity and selectivity [79, 80, 81]. A comparison of GC–MS and GC×GC–MS has also been made in this application. Abel et al. explored the sensitivity of various GC techniques in detecting ignitable liquids in forensic fire debris analysis, specifically focusing on gasoline and diesel [74]. They systematically tested the limit of identification (LOI) for three different GC–MS configurations (GC–MS, GC–TOFMS, and GC×GC–TOFMS) using known amounts of gasoline and diesel, both in pure form and mixed with pyrolysis products. Their results demonstrated that GC×GC–TOFMS offers significantly greater sensitivity, especially for identifying diesel, highlighting the impact this technique could offer in forensic investigations. The authors also emphasized the need for further research to establish the appropriate sensitivity levels that minimize misinterpretation while maintaining the necessary information for fire investigations [74]. Nizio et al. also demonstrated the benefit of using GC×GC for kerosene analysis over 1D GC (Figure 4) [82]. Development of detection methods and displays of data for evidence presentation demonstrate an advancement of this field toward forensic readiness.

Standardization of methods has been outlined by multiple manuscripts. Boegelsack et al. created a comprehensive workflow for developing a robust and efficient analytical method using flow‐modulated GC×GC–MS to identify ILRs in fire debris [35]. The authors highlighted the challenges of traditional methods in dealing with complex matrices and overlapping peaks, emphasizing the advantages of GC×GC in achieving higher resolution and separation. Their research utilized design of experiments (DoE) and response surface methodology (RSM) to optimize variables, enhancing the sensitivity, selectivity, and accuracy of ILR identification [35]. In a subsequent study, a new retention index (RI) system, the “KLI” system, which combines two established RI systems—the Kovats index (K) for the first dimension and the Lee index (L) for the second dimension—within GC×GC was proposed [36]. The KLI system showed strong correlations with predicted values and existing RI systems and demonstrated high robustness across varying analyte concentrations and operational settings [36]. Both methods demonstrated successful classification of ILRs based on ASTM E1618 standards, showcasing a potential for wildfire investigations.

Further studies continued to refine GC×GC–MS methods, with a focus on increasing peak capacity and characterizing specific ILR classes, such as white spirits [39, 82]. The concept of Local Ion Signatures (LIS) was also further developed, improving ILR detection and classification [37, 38, 40]. Pandohee et al. used GC×GC‐FID to analyze various petrochemicals, including gasoline, kerosene, diesel, and turpentine, in both fresh and weathered states [83]. They demonstrated the technique's effectiveness by visually comparing the chemical fingerprints of different fuels and using principal component analysis (PCA) to differentiate between fuel types and their weathering stages.

Nizio and Forbes developed methods for collecting and analyzing ILRs from burnt human remains, addressing challenges in sample preparation and analysis [40]. They proposed using dynamic headspace sampling with GC×GC–TOFMS, showing its effectiveness in detecting gasoline residues from burnt porcine remains and highlighting its potential in forensic applications. Kates et al. examined the use of GC×GC–TOFMS for analyzing ILRs in wildfire debris, focusing on 164 samples flagged by canine detection units [84]. Their results indicated that traditional GC–MS methods often struggle to accurately identify ILRs in complex wildfire debris due to interference from naturally occurring compounds and pyrolysis by‐products [84]. In contrast, GC×GC–TOFMS provided superior separation capabilities and lower detection limits, allowing for clearer and more definitive ILR identification.

Boegelsack et al. investigated cross‐contamination of ILR samples on different matrices, such as gravel, soil, and wood, in controlled conditions [85, 86]. In both studies, GC×GC–TOFMS played a critical role in detecting and analyzing cross‐contamination of ILRs in fire debris samples. In the first study, GC×GC–TOFMS enabled highly sensitive detection of cross‐contamination across various matrices, identifying even trace amounts of transferred ILRs and providing detailed insights into matrix‐specific interactions and potential false positives [85]. In the second study, GC×GC–TOFMS was essential for comparing different packaging materials’ effectiveness in preventing ILR contamination. It enabled the precise measurement of ILR transfer and interference from packaging materials, supporting the identification of optimal storage and transport methods to maintain forensic sample integrity [86]. In both cases, GC×GC's HR and chemometric capabilities allowed for accurate differentiation of genuine ILR samples from those affected by cross‐contamination.

There is a clear need for rigorous inter‐laboratory validation to ensure the reliability and comparability of results across different laboratories [36, 38, 74]. Boegelsack et al. emphasize the challenges posed by the lack of standardized methods and protocols for GC×GC–MS in fire debris analysis, noting how this lack of standardization can complicate the presentation of findings in court [36]. Multiple studies have been discussed that demonstrate the effectiveness and superiority of GC×GC–MS over GC–MS or GC‐FID methods. Although some studies represent TRL 3 readiness, more supporting data for the identification of ILRs using the same protocol, including data processing and analysis, are necessary to complete a TRL of 2. The lack of error rates and standardization of methods hinders the advancement of this application to a full TRL of 3. Future research should focus on identifying figures of merit and relaying results of inter‐laboratory trials to progress this application.

A consistent theme across the literature is the importance of analyst expertise and training in fire debris analysis. The complexity of the analysis, the presence of interfering products, and the need for accurate pattern recognition all necessitate a high level of expertise, which is relevant especially to voir dire and the Mohan criteria. Ongoing education, training, and certification are critical to maintaining and improving the quality of fire debris analysis. Moreover, the application of advanced techniques, such as GC×GC–TOFMS, requires standardized protocols, careful data interpretation, and effective communication between investigators and analysts to ensure accuracy and reliability in fire debris analysis.

## 8 Odor Decomposition

The analysis of volatile organic compounds (VOCs) originating from human remains and human analogs has been of interest in the field of forensic taphonomy since the first studies in 2004 [87]. In a forensic context, determining the progression of decomposition often assists in estimating postmortem interval (PMI) and in establishing both spatial and temporal events associated with a case involving a decedent. There is great interest in using the chemical analysis of VOCs for several purposes, including to understand the mechanisms behind human decomposition, how insects are attracted to remains, and the alert process of scent detection canines when searching for human remains, as well as to develop possible portable sensors for search and recovery. Early works focused on defining the VOCs found from decomposing remains using GC–MS from 2004 until 2012 [87, 88, 89, 90, 91, 92, 93, 94, 95, 96, 97, 98].

From 2012 until the present, there has been a large influx in research leveraging the increased peak capacity of GC×GC–MS to improve the comprehensive characterization of decomposition odor. Although early GC–MS studies predicted hundreds or thousands of compounds in decomposition odor, the use of GC×GC–MS drastically improved the ability of the field to document this list of VOCs more confidently and to semi‐quantitatively monitor trace‐level changes in each VOC component within the overall profile. The early proof‐of‐concept work on using GC×GC–TOFMS for decomposition odor profiling in 2012–2013 conducted by Dekeirsschieter et al., Brasseur et al., Focant et al., and Stadler et al. supported the potential of this technique toward improving foundational knowledge of the broad range of VOCs released during cadaveric decomposition in human analogs, specifically using pig carcasses (*Sus scrofa domesticus*) [23, 91, 99, 100]. Beyond these earliest research articles demonstrating foundational proof‐of‐concept and some promising repeatability in profile findings, it became possible to compare more subtle differences in profiles, especially in fieldwork trials and other areas that required differentiation of samples based on all possible components in a nontargeted approach. Forbes and Perrault demonstrated differences in air and soil during varied seasons using human analogs [101, 102]. A comparison of GC–MS and GC×GC–MS was performed by Perrault et al. [8], published nearly simultaneously with studies investigating data processing approaches [103], varied scenarios involving the specific placement, movement, or climate for decomposing remains [22, 53, 104, 105], the first study using GC×GC–MS on samples from human cadavers [106], and the use of advanced HRTOFMS [107]. Foundational knowledge from GC–MS and GC×GC–MS studies was expanded greatly during the early 2010s to support growing interest in this field.

Beyond these new, interesting applications, data integrity became an increasing focus. In 2015, Perrault et al. published research focusing on background subtraction, the use of appropriate controls, replication requirements, and data handling strategies to serve as a foundation for decreasing variation in analytical results between studies with the aim of increasing confidence and decreasing error in the field [104]. Most of these guidelines have been considered in following fieldwork studies. For example, using these background subtraction and control design strategies, Armstrong et al. focused on field trials to characterize VOCs in the early postmortem period, and Forbes et al. focused on the release of odor at belowground graves using soil probing [108, 109]. These replication studies begin to address higher level TRL topics as this application moves past the proof‐of‐concept stage to more robust analytical development.

Although earlier studies were conducted with pig carcasses as analogs for human decomposition, several later studies used human remains or tissues to validate the approach of GC×GC–MS use to analyze decomposition odor. Some studies have investigated the ability to withdraw gases from cadavers at autopsy [110, 111], whereas other studies focused on using human tissues individually [26, 112, 113]. There has also been one study conducted on a real forensic case sample [25]. Knobel et al. conducted a study focusing on the placement of both human remains and pig carcasses in the same environment under the same conditions to more effectively understand the differences between human and animal remains [28]. More recently, seasonal variation on whole human cadavers [114], the use of amputated limbs to represent whole human cadavers [24], and the identification of VOCs from human remains in water [115] have been investigated. VOC profiling of human remains from cadavers and human bones has also been used to aid in the training of human remains detection canines with applications for search and rescue missions [116, 117]. The application of human remains VOC profiling using established methods from pig carcass samples progresses the readiness of this field toward appropriate validation for forensic laboratory use.

On the basis of the number of studies being conducted with GC×GC–MS in this field, the technique has certainly reached general acceptance as a research tool that is valuable and becoming the gold standard for continued studies. Although decomposition odor has been presented in forensic cases in the infancy of the field, there is now a large body of literature, including both GC–MS and GC×GC–MS, that provides improved validity of this type of profiling to improve our understanding of forensic taphonomy [118]. Regarding Daubert criteria, the technique has been peer‐reviewed and published as well as tested against conventional techniques, whereas studies have also been conducted demonstrating best practices for data integrity. However, there is still a large gap in understanding the error rates associated with using GC–MS or GC×GC–MS data toward forensic casework. For example, there is an absence of studies that characterize decomposition odor alongside other distractor odors to improve our understanding of how specific the profile detected must be to definitively conclude the presence of decomposition odor (e.g., as opposed to garbage, animal remains, or other decomposing organic material). On the basis of some missing components of error rates and acceptable limits, the absence of inter‐laboratory comparison on the same samples, as well as the lack of a validated or standardized method that could be implemented in casework, this application reaches the beginning of a TRL 3. Additional work on error rates, inter‐laboratory trials, as well as the development of field‐specific standards would serve to assist in the use of GC×GC–MS as a valuable tool that can be immediately adopted in forensic casework.

## 9 Oil Spills

The use of GC×GC for oil spill forensics is far from a new proof‐of‐concept study. The general development of GC×GC technology and applications originated through the study of complex petroleum substances [6]. The field of oil spill forensics is a subset of petroleum chemistry that aims to characterize class distinctions and recalcitrant biomarkers within petroleum products for source identification and time‐after‐release analysis in the case of forensically related industrial spillage. There is extensive background research on petroleum sampling, class characterization, and data processing in the 1D GC sphere, as well as using GC×GC for petroleum analysis [119]. Oil spill forensic research focuses on the changes in chemical composition of the oil after it is released into its environment. These changes occur via evaporation, degradation, weathering, and photooxidation mechanisms, which can vary based on climate conditions, type of released oil, and time in the environment [81, 120, 121]. Monitoring these changes using advanced chromatographic techniques gives researchers a more complete picture of these natural processes and informs prediction modeling and tracking for oil spill investigations.

The first application using GC×GC‐FID to identify a source of a marine oil spill was conducted on spillage from a marine vessel compared to two potential diesel sources with successful determination of the diesel source [122]. Other early GC×GC‐FID studies focused on identifying biomarkers within petroleum that could be used in spill analysis for source identification [123] while also comparing results of degradation processes to a previously studied spill that used traditional GC–MS methods [124]. Important early studies established GC×GC‐FID as a powerful tool for this type of petroleum analysis by comparing 1D GC results to GC×GC results of decades‐old spilled oil from the *Florida* barge spill in 1969 and Exxon Valdez spill in 1988 [124, 125]. With greater resolving power and peak capacity for investigating the unresolved complex mixture (UCM) that often arises in 1D GC analysis of petroleum, it is no surprise that GC×GC was established as a superior technique for this application.

Sequential research focused on prediction of weathering, degradation, and evaporation patterns of hydrocarbon content for tracking an oil spill. Many studies used the Deepwater Horizon spill of 2010 as a case study for chemical composition analysis after a spill [47, 50, 126, 127, 128, 129, 130, 131]. Of 19 studies that performed analysis on real oil spills, 8 were conducted on spillage from Deepwater Horizon. Investigation into the weathering patterns of spilled oil from the barge *Bouchard 120* and Deepwater Horizon provided insight into the weathering of oil in the environment. Model predictions for weathering patterns matched observed patterns of the *Bouchard* spill [42], whereas recommendations for updating models to include oxyhydrocarbon mass of weathered oil were suggested after probing of the Deepwater Horizon spill [127]. Field studies on real oil spill cases provide realistic insight into the compositional changes of oil in a variety of environmental conditions.

Oil spilled in the natural environment is degraded by biotic and abiotic factors, including microbes and sunlight. Microbial biodegradation of oil was first studied by DeMello et al. using stored samples from the *Bouchard 65* spill in 1974 in an ex‐situ laboratory experiment [132]. Fatty acid methyl ester (FAME) degradation was found to stabilize with biodegradation, which the authors concluded would impact transport and weathering processes of oil for forensic analysis [132]. Gros et al. expanded upon understanding of biodegradation using oil from Deepwater Horizon; the stepwise degradation process they found supported previous 1D GC research and was used to create a new biodegradation index for oil weathering [47]. Lemkau et al. determined a biomarker ratio of C_18_/phytane for biodegradation rate from oil of the M/V *Coco Busan* in 2007 [133]. Initial probing into biodegradation using GC×GC shows promise for future use as an analysis tool for tracking spills. Additionally, four manuscripts have been identified by this review in which photodegradation of oil has been studied for forensic identification [130, 133, 134, 135]. A biomarker ratio of benz[a]anthracene/chrysene was found to indicate photodegradation rates and could be useful in identifying time since spill in an investigation process [133]. Ward et al. also investigated time since spill using C_18_/phytane ratios [135]. Patterns of photodegradation influenced by aromaticity were identified by Radović et al. [130]. Snyder et al. compared photodegradation rates of spilled oil to oil from a natural seep to identify differences in the weathering processes of the two sources for differentiation of oil origins [134]. Although these studies demonstrate the capability of GC×GC to aid in forensic analysis of bio‐ and photodegradation of oil, supporting data and validation methods must be developed for use of these methods in a forensic context.

Evaporation is a third alternative fate of oil that has been spilled. Arey et al. differentiated evaporation from dissolution processes using hydrocarbon mass loss as an indicator and then compared those predictions to a working model for updated accuracy [41, 42]. Gros et al. performed a novel study of evaporation of hydrocarbons during the first day of an intentional, controlled oil spill to update prediction models of oil spill movement and tracking [136]. The evaluation of evaporation patterns informs prediction and early assessment of oil spill tracking and timelines.

Chemometric techniques for data processing and analysis were simultaneously developed while oil depletion processes in the environment were studied. Visualization techniques like difference, ratio, and addition chromatograms were explored to physically present data results [120]. Mass loss tables (MLTs) for evaporation predictions based on hydrocarbon volatility and peak topography maps to focus chromatographic analysis on biomarker regions of interest were developed for easier GC×GC data interpretation [41, 42, 131]. Development of chemometric techniques for understanding GC×GC data goes hand in hand with the development and standardization of sampling and chromatographic methods.

Other petroleum forms have been evaluated with GC×GC for source identification with forensic applications. This technique was applied by Jaramillo and Dorman and Piotrowski et al. to hydraulic oil contamination from fracking processes [137, 138]. The crude form of petroleum, bitumen, was evaluated for fingerprint biomarkers in a proof‐of‐concept study by Sojinu and successfully compared to water and soil samples of potential mining source locations [139]. These proof‐of‐concept studies build upon known petroleum chemistry but are the first of their kind to perform analysis on their respective sample type using GC×GC.

Research in over 30 manuscripts published from 1999 to the 2020s established oil spill tracing as a forensic application that benefits from GC×GC. This application has sufficient supporting data for the development of theories and research as defined by a technology readiness of 2 and meets the Frye and Daubert criteria for general acceptance but does not fill all criteria for the four‐part validation process of Daubert or the Federal Rule of 702. Measurements of uncertainty and inter‐laboratory validation should be the next areas of focus for this application to complete a TRL of 3 and advance this application on the scale of usability for forensic laboratories.

## 10 Summary and Outlook

**FIGURE 5:**
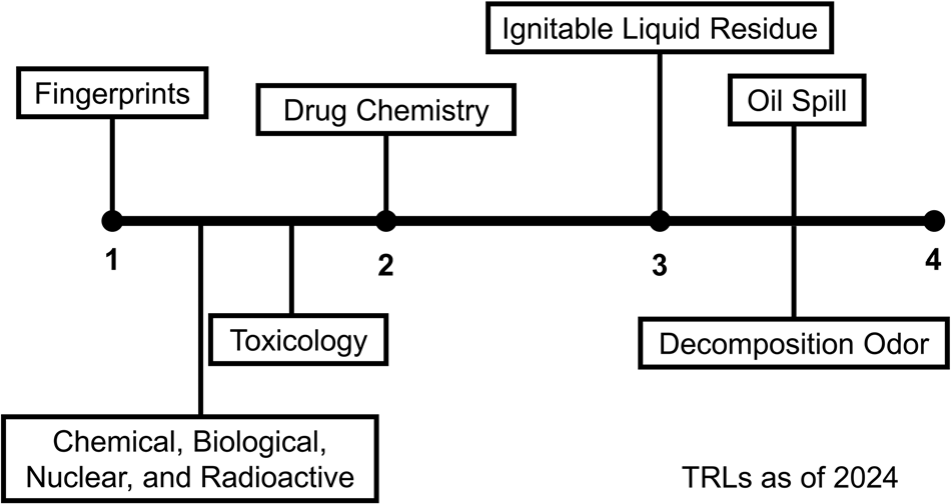
TRL comparison of seven forensic chemistry applications using comprehensive two‐dimensional gas chromatography in 2024. TRL, technology readiness level.

Although GC×GC is not a new concept as of 2024, its application in the field of forensic chemistry has significantly expanded over the past two decades. The seven forensic chemistry applications discussed in this review are categorized in various stages of development for use in the forensic laboratory. Although not ready for routine analysis currently, all applications require research in the areas of intra‐ and inter‐laboratory studies, development of figures of merit, and increased replication studies to move from a developmental phase of research to an established and “gold standard” method. However, it is of note that current research in all application areas is being conducted on commercially available instrumentation. This is a large step in the standardization of chromatographic methods when the instrument itself has already undergone a degree of standardization through commercial manufacturing. Research applications, such as fingerprints, CBNR, and toxicology, are in early stages of development with the establishment of basic theory and research but lacking development, supporting data, or any higher TRL level figure of merit or validation experiments (Figure 5). Drug chemistry analysis using GC×GC is slightly further developed at a TRL of 2, moving past proof‐of‐concept studies but requiring replication and validation for further progression (Figure 5). The application of ILR for arson investigation has shown development within the application, especially relating to data analysis, but lacks the replication of supporting data to fully move into a TRL of 3. Replication and repeatability studies allow oil spill forensics and decomposition odor applications to be categorized as higher TRLs (Figure 5). The few inter‐laboratory studies conducted have shown promise in the ability to further develop validation through figures of merit. The development of chemometric techniques for analyzing and displaying data has been more heavily explored in recent years, which could also reduce some of the challenges associated with expert user necessity for GC×GC output interpretation.

Although there are studies in every application area that compare GC×GC to GC using the same methods, few are direct comparison studies using methods from the Organization of Scientific Area Committees for Forensic Science (OSAC) or ASTM. The optimization, validation, and replication of OSAC and ASTM methods are a critical component of technology readiness because standardization is a major development goal for new analytical technologies to be adopted into forensic laboratories. Within this process is the need for these methods to be translated from established ASTM methods or created into fully new methods. In addition to standardization and validation of sampling and chromatographic methods, the same standardization must occur within the data analysis sphere in order to use these techniques within a forensic laboratory. Although not specifically mentioned in any current works in the field of forensic GC×GC analysis, it is also likely that an increased emphasis on green chemistry may also come into play when looking forward to future standardized methods since sustainability is becoming a major focus for routine laboratories with high throughput.

General acceptance of a technology or forensic method is important in courtroom admittance, as seen as the basis for over 100 years of scientific evidence admittance. This principle is seen in the Frye and Daubert criteria, on which Federal Rule of 702 is based (Table 1). On the basis of the number of published and reviewed studies in the three higher TRL applications of ILR, oil spill, and decomposition odor, there is general acceptance in their respective scientific communities for the use of this new technology within the forensic sphere (Figure 3). The other four applications discussed in this review comparatively have less than half the number of publications as the two leading applications. Depending on their TRL, general acceptance could be established in subsequent years. TRL 1 applications have further to go for the research to be considered sufficient for general acceptance. Another courtroom concept touched on by authors of current literature addresses the need for an increase in the expertise of the forensic workforce for a new technique such as GC×GC to be adopted into routine forensic testing and laboratory spaces. The Mohan criteria in Canada and the US Federal Rule of 702 explicitly address the scientific expert's role, in this case the forensic analyst, in testimony. Both rules cover themes relating to the qualification and appropriate application of methods to the forensic evidence in question. The importance of a competent workforce of analytical chemists in forensics is reinforced by these requirements and should not be neglected when considering training and implementation from an academic standpoint as well as a forensic chemistry lens.

Researchers in the field of forensic chemistry have demonstrated the value of increased peak capacity and sensitivity from GC×GC and the positive outcome it has in increasing understanding of potential forensic evidence. Although some applications are still in their infancy in regard to exploration with GC×GC, more mature and developed research in oil spill forensics and decomposition odor indicates a push for readiness in routine forensic laboratories. Early readiness stage applications can learn from this TRL progression to inform workflows and determine the most valuable next research steps in terms of study design and validation. Although the research indicates that GC×GC might not be ready for implementation in forensic laboratories across all disciplines currently in 2024, there is great potential for its use and admission within the court system in the future.

## Conflicts of Interest

The authors declare no conflicts of interest.

## Data Availability

The data that support the findings of this study are available from the corresponding author upon reasonable request.
